# Divergent kinase WNG1 is regulated by phosphorylation of an atypical activation sub-domain

**DOI:** 10.1042/BCJ20220076

**Published:** 2022-09-16

**Authors:** Pravin S. Dewangan, Tsebaot G. Beraki, E. Ariana Paiz, Delia Appiah Mensah, Zhe Chen, Michael L. Reese

**Affiliations:** 1 Department of Pharmacology, University of Texas, Southwestern Medical Center, Dallas, TX, U.S.A.; 2 Honors College, University of Texas at Dallas, Richardson, TX, U.S.A.; 3 Department of Biophysics, University of Texas, Southwestern Medical Center, Dallas, TX, U.S.A.; 4 Department of Biochemistry, University of Texas, Southwestern Medical Center, Dallas, TX, U.S.A.

**Keywords:** apicomplexa, cellular secretion, phosphorylation, pseudokinases

## Abstract

Apicomplexan parasites like *Toxoplasma gondii* grow and replicate within a specialized organelle called the parasitophorous vacuole. The vacuole is decorated with parasite proteins that integrate into the membrane after trafficking through the parasite secretory system as soluble, chaperoned complexes. A regulator of this process is an atypical protein kinase called WNG1. Phosphorylation by WNG1 appears to serve as a switch for membrane integration. However, like its substrates, WNG1 is secreted from the parasite dense granules, and its activity must, therefore, be tightly regulated until the correct membrane is encountered. Here, we demonstrate that, while another member of the WNG family can adopt multiple multimeric states, WNG1 is monomeric and therefore not regulated by multimerization. Instead, we identify two phosphosites on WNG1 that are required for its kinase activity. Using a combination of *in vitro* biochemistry and structural modeling, we identify basic residues that are also required for WNG1 activity and appear to recognize the activating phosphosites. Among these coordinating residues are the ‘HRD’ Arg, which recognizes activation loop phosphorylation in canonical kinases. WNG1, however, is not phosphorylated on its activation loop, but rather on atypical phosphosites on its C-lobe. We propose a simple model in which WNG1 is activated by increasing ATP concentration above a critical threshold once the kinase traffics to the parasitophorous vacuole.

## Introduction

Protein kinases are the largest protein family in eukaryotes and regulate diverse cellular functions by phosphorylation [1]. To maintain the fidelity of signaling, many kinases must be activated before they can efficiently carry out phosphoryl transfer [2–9]. Indeed, uncontrolled kinase activity is often pathogenic and is, therefore, at the heart of many diseases [10–18]. The two most common mechanisms by which kinase activities are regulated are multimerization [19–21] and phosphorylation on the kinase ‘activation loop’ [22–25]. While the regulation of canonical protein kinases is now well understood, a subset of protein kinases have many atypical motifs and therefore function through unusual mechanisms [26–29].

Apicomplexan parasites such as *Toxoplasma gondii* encode an unusually high number of pseudokinases and atypical kinases [30,31], the majority of which are secreted into the host cell during invasion [30,32,33]. Among these kinases is a family that lacks the Glycine-rich loop that is required for kinase activity in all other kinases [34–36]. Nevertheless, members of these ‘With-No-Gly-loop’ (WNG) kinases are still able to robustly catalyze phosphoryl transfer using a reorganized kinase structure [34]. The most highly conserved member of the family, WNG1, is secreted into the *T. gondii* parasitophorous vacuole (PV), the organelle in which the parasite grows and replicates within an infected host cell. WNG1 phosphorylates many other secreted proteins, and appears to regulate their insertion into the PV membrane [34]. Notably, these PV-resident membrane proteins are trafficked through the parasite Golgi and secretory granules as soluble entities [37], and integrate only into the correct membrane. Because the kinase is secreted from the very same organelles as its substrates, we reason that WNG1 must be maintained inactive prior to secretion to ensure the correct trafficking of its substrates.

In the present work, we sought to determine the mechanism by which WNG1 activity is regulated. We found that another WNG family member, BPK1, can exist in different multimeric states. WNG1, however, does not multimerize in solution and is, therefore, unlikely to be regulated in this manner. Instead, we found that WNG1 can autophosphorylate two residues in its C-lobe distinct from the activation loop, and that these sites are required for WNG1 activity. We also identified a series of basic residues that are also required for WNG1 activity and therefore appear to be recognizing the activating phosphosites. These apparent regulatory sites are distinct from those found on more typical kinases, highlighting the structural and biochemical divergence of the WNG family.

## Results

### WNG1 does not oligomerize in solution

One mechanism by which kinase activity can be regulated is through changes in the multimerization state. We had previously crystallized a WNG family member, the pseudokinase BPK1, and found that it forms a homohexameric assembly in all crystal forms we obtained (Supplementary Figure S1). Notably, the BPK1 pseudoactive sites are oriented into the closed hexamer. SAXS analysis also revealed that BPK1 appears to adopt a tetrameric state in solution that is distinct from the hexameric arrangement found in crystals (Supplementary Figure S1 and Supplementary Table S1). BPK1 is a catalytically inactive pseudokinase and shares only ∼30% sequence identity with active family members such as WNG1. Nevertheless, because the closed hexamer state of BPK1 would be unable to interact with the substrate, we reasoned that such a multimeric state may be an inhibited state conserved among WNG family members.

**Figure 1.**
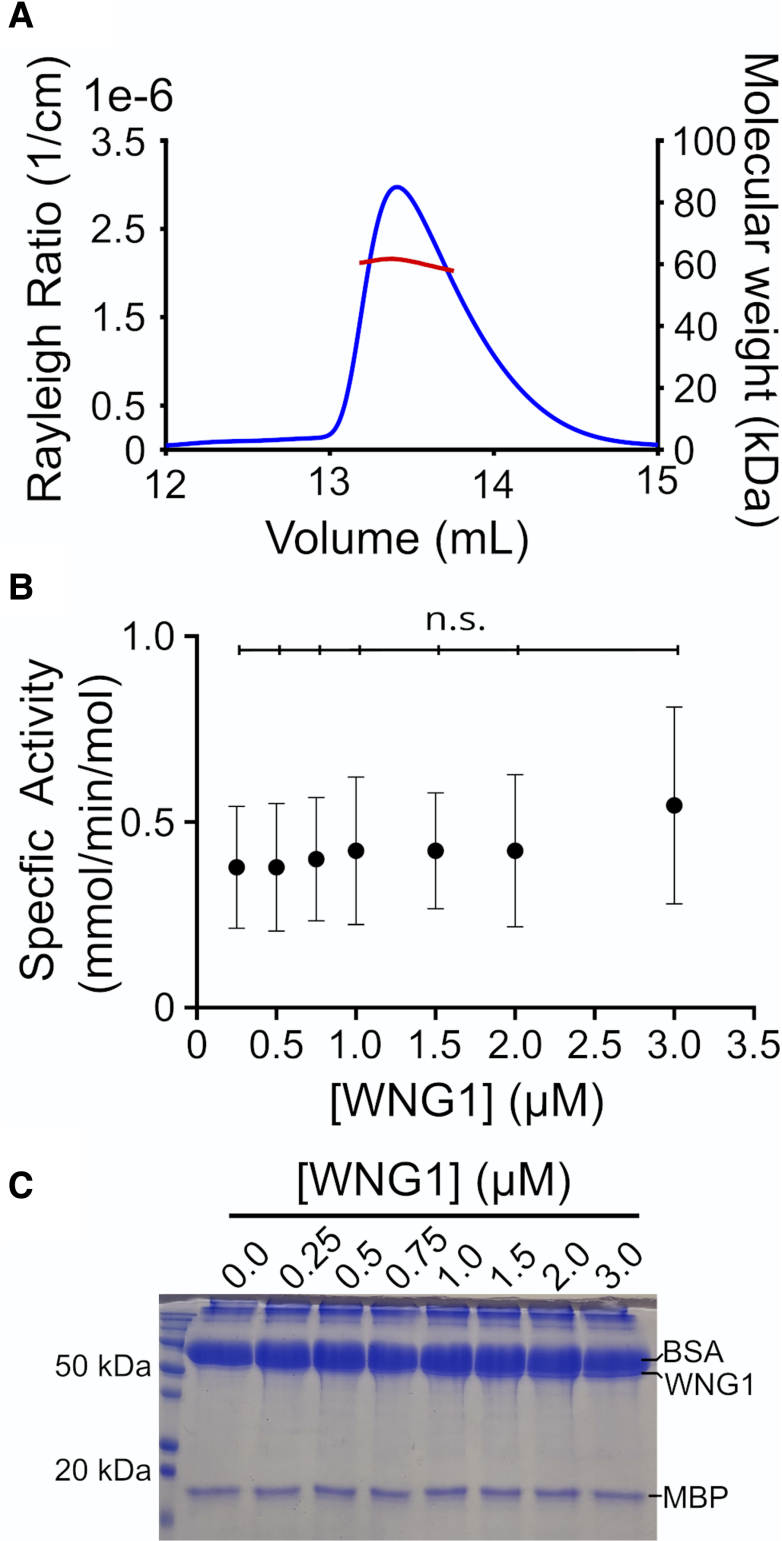
WNG1 is a monomer and its activity is concentration independent. (**A**) Size exclusion chromatography coupled with multi-angle light scattering (SEC-MALS) profile for SUMO-WNG1_265–591_ using a Superdex 200 10/30 Increase column. The blue line represents the scattering curve and the red line represents the molecular mass distribution. (**B**) Kinase activity measurement for SUMO-WNG1_265–591_ at the indicated enzyme concentrations. Significance was determined by One-way ANOVA followed by Tukey's test; n.s., not significant. (**C**) A representative SDS–PAGE gel for the kinase assay (**B**) is shown below the graph. The lanes indicate the concentration of WNG1 (in μM) used in the kinase assays.

We, therefore, sought to determine if the WNG family member and an active kinase, WNG1, was also able to adopt similar multimerization states. We bacterially expressed the WNG1 kinase domain (residues 265–591) as a His_6_-SUMO fusion and purified the protein. We coupled size exclusion chromatography with multi-angle light scattering to estimate the molecular mass of the purified WNG1 kinase (Figure 1A). This analysis indicated that SUMO-WNG1 has a radius of gyration consistent with a 60 kDa globular protein, very close to the predicted 51 kDa of the construct. These data, therefore, suggest that SUMO-WNG1 is monomeric in solution, and, unlike BPK1, does not appear to form tightly associated multimers. However, we could not rule out that WNG1 formed transient multimers that may affect kinase activity. Such regulation of enzymatic activity by multimerization would be evident as a concentration-dependence on kinase specific activity (i.e. cooperativity). We, therefore, assessed WNG1 kinase activity at a range of WNG1 concentrations (0.25–3 μM) using myelin basic protein (MBP) as a substrate. WNG1 specific activity was independent of kinase concentration (Figure 1B,C) within the concentration range tested, leading us to conclude that WNG1 activation is not dependent on multimerization in solution.

### WNG1 autophosphorylates residues not associated with regulation of other kinases

**Figure 2.**
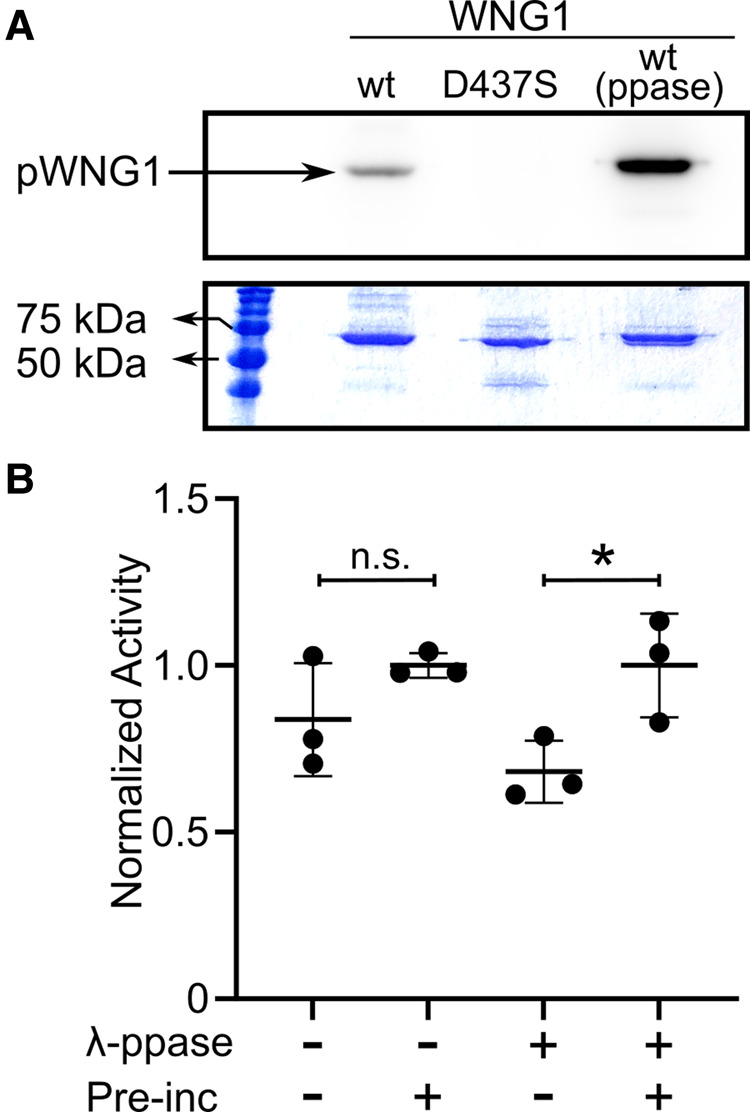
WNG1 is activated by autophosphorylation. (**A**) Autoradiogram showing autophosphorylation of wild-type WNG1 (coexpressed ± lambda phosphatase) in a 30 min reaction. The kinase dead D437S mutant is shown as a negative control. Note that dephosphorylated WNG1 is more highly autophosphorylated than the protein expressed without phosphatase when incubated for longer periods, likely due to a larger number of available phosphosites, Corresponding SDS–PAGE gel is shown for reference. (**B**) WNG1 kinase co-expressed with lambda phosphatase (right columns) required a 5 min pre-incubation with cold ATP to reach full activity against MBP (1 min incubation with [γ-^32^P]-ATP). Samples were normalized to the ‘pre-incubated’ sample of each ± phosphatase. Significance was determined by One-way ANOVA followed by Šidák's multiple comparisons test; (*, *P* < 0.05; n.s., not significant).

After ruling out multimerization of WNG1 as a likely regulatory mechanism, we tested whether WNG1 may be activated by phosphorylation. Recombinant WNG1 robustly autophosphorylates (Figure 2A), suggesting it may autoactivate via this mechanism. Consistent with this idea, kinase that was coexpressed with lambda phosphatase required pre-incubation with ATP to obtain full activity against MBP (Figure 2B). Note, however, that WNG1 appears to autoactivate very quickly, as a significant difference in MBP phosphorylation between the pre-incubated and not pre-incubated phosphatase-treated kinase was only observed when incubation times with MBP were ≤2 min.

**Figure 3.**
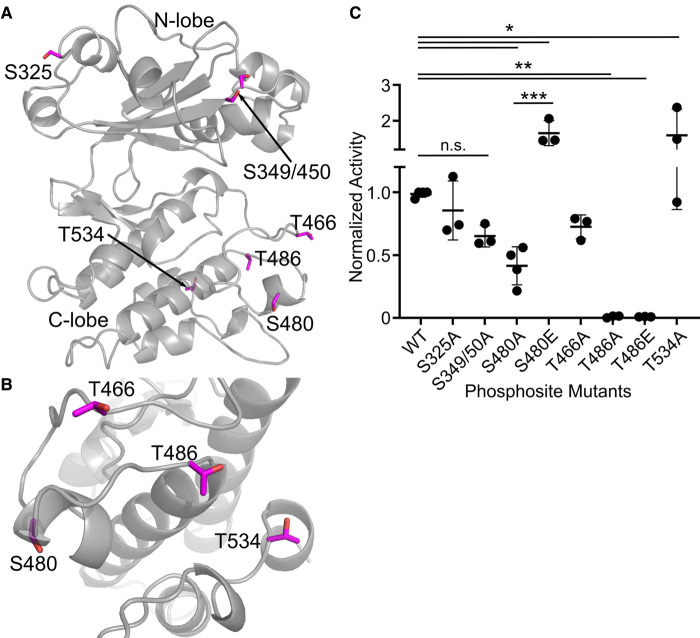
WNG1 autophosphorylation at specific residues is essential for its activity. (**A**) WNG1 homology model generated from BPK1 structure is represented in gray color. The serine/threonine residues identified from the mass spectrometry are shown as sticks in magenta color. (**B**) Rotated (−90°) and zoomed view of the WNG1 C-lobe serine/threonine phosphosites (magenta sticks). (**C**) Kinase activity of WNG1 protein with non-phosphorylatable (Ala) mutants of identified phosphosites and phosphomimatic T486E and S480E. Significance was determined by One-way ANOVA followed by Dunnett's multiple comparisons test (for comparison to wild-type control). Sidak's multiple comparisons test was used to evaluate S480A vs S480E and T486A vs T486E. (*P < 0.05; **P < 0.01; ***P < 0.001; n.s., not significant).

Given the apparent activating autophosphorylation, we performed mass spectrometry to compare the phosphosites found on recombinantly purified wild-type versus kinase dead (D437S) WNG1. From these data, we identified residues Ser325, Ser349, Ser350, Ser480, Thr486 and Thr534 as robustly phosphorylated. Since none of the residues we identified as phosphorylated on WNG1 are present on sites conventionally thought of as regulating kinase activity (Figure 3A) we wanted to know if their phosphorylation was essential for WNG1 kinase activity. The first three sites are located in the N-lobe while the latter three are located in the C-lobe of WNG1 (Figure 3A,B). Strikingly, none of these residues are within the WNG1 activation loop, and to our knowledge, are not at sites that have been associated with activation of other kinases [23,25,28]. To test whether phosphorylation of these phosphosites is essential for WNG1 activity, we mutated each of the identified phosphosites to Ala. Notably, WNG1 has one phosphorylatable residue, Thr466, in its activation loop (Figure 3A,B), though we did not identify it as phosphorylated in our mass spectrometry data. However, we had previously shown that WNG1 requires its HRD Arg436 for activity [34], a site which normally recognizes phosphorylated Ser/Thr in a canonical kinase activation loop [23,25]. To confirm Thr466 is not required for WNG1 activity, we also created a T466A mutant.

We evaluated the *in vitro* kinase activities of each mutant. Mutation of the three N-lobe sites did not significantly affect kinase activity (Figure 3C). Thus, phosphorylation in the N-lobe of WNG1 appears dispensable for its kinase activity. We also observed no significant change in activity upon mutation of Thr466, confirming that WNG1 does not require phosphorylation on its activation loop for activity. Somewhat surprisingly, mutation of Thr534 increased the specific activity of the kinase (Figure 3C). On the other hand, the Ser480 mutant had ∼40% activity of wild-type WNG1 (Figure 3C). Most strikingly, mutation of Thr486 rendered WNG1 completely inactive. Protein–protein interactions driven by phosphorylation can sometimes be rescued by mutation of phosphosites to a negatively charged residue. We, therefore, tested whether mutation of Ser480 or Thr486 to Glu restored WNG1 kinase activity. Consistent with a role as a regulatory phosphosite, we found that S480E showed significantly higher activity towards MBP than the uncharged Ala mutant (Figure 3C). Mutation of T486E, however, did not restore activity. Nevertheless, these data, suggest that WNG1 requires phosphorylation on non-canonical sites on its C-lobe for kinase activity.

### Basic residues in proximity of T486 are essential for WNG1 activity

**Figure 4.**
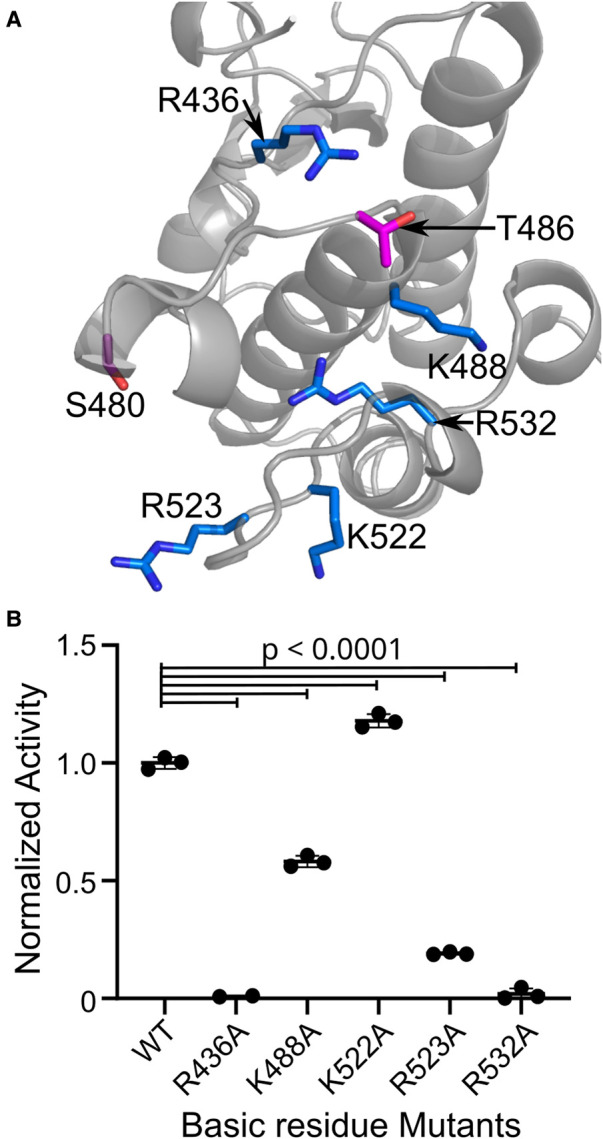
Basic residues in proximity of the phosphosites are important for WNG1 kinase activity. (**A**) C-lobe of the WNG1 homology model showing the basic amino acids (represented as blue sticks) within 4 Å of S480 and T486 (represented as magenta sticks). (**B**) Kinase activity measurement of the WNG1 protein with alanine mutation at R436, K488, K522, R523 and R532. Significance was determined by One-way ANOVA followed by Dunnett's multiple comparisons test.

**Figure 5.**
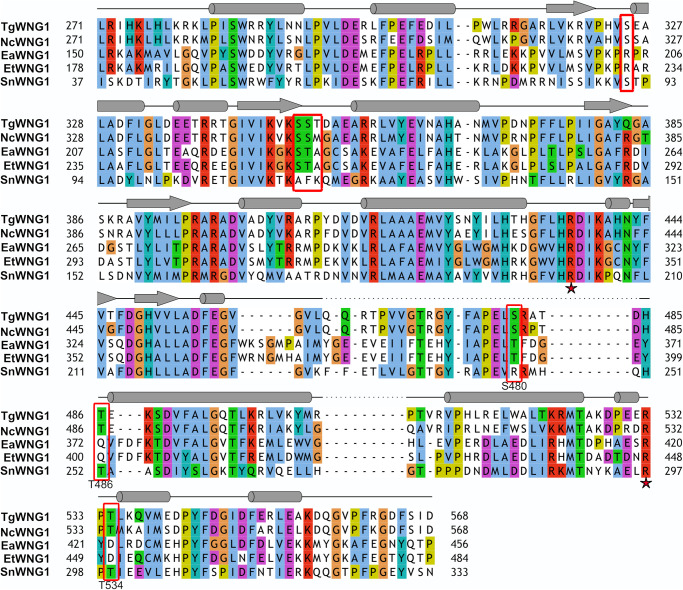
Sequence alignment of WNG1 orthologs. Sequence alignment of the *Toxoplasma* WNG1 kinase domain and its orthologs from selected coccidian parasites. The residues that are autophososphorylated in the recombinant protein are highlighted in red rectangles. The Arg residues R436 and R532 that are proximal to identified phosphosites and are essential for WNG1 activity are marked with red star. The predicted secondary structure is represented on top of the corresponding WNG1 sequence; helices as cylinders and β-sheet is represented by arrows (Tg, *Toxoplasma gondii*; Nc, *Neospora caninum*; Ea, *Eimeria acervulina*; Et, *Eimeria tenella*; Sn, *Sarcocystis neurona*).

As strongly electronegative residues, phosphosites are typically recognized by basic patches of Lys and Arg [23–25]. We, therefore, sought to identify basic residues that may recognize the WNG1 activating phosphosites Ser480 and Thr486. We generated a homology model of the WNG1 structure using BPK1 as a template. We used this model to identify basic residues with side chains within 4 Å of Thr486 or Ser480 (Figure 4). Consistent with its published mutant effect on kinase activity [34], we found that the HRD Arg436 is within 4 Å of Thr486, and may, therefore, recognize its phospho-state. In addition to Arg436, four other Arg/Lys are within range of the phosphosites to potentially form salt bridges (Figure 4A). Notably, these basic residues are each conserved in WNG1 kinases from other species (Figure 5). To test if these basic residues were essential for kinase activity, we mutated each individually to Ala. We expressed and purified each mutant protein and tested its *in vitro* kinase activity. Mutants of Arg436 and Arg532 were completely inactive (Figure 4B), consistent with potential roles in coordinating a phosphorylated Thr486. However, we cannot rule out that reduced thermal stability of the R436A mutant protein affected its activity (Supplementary Figure S2). Lys488 is also nearby Thr486, though its mutation to Ala resulted in only a minor 40% reduction in activity (Figure 4B). Lys522 and Arg523 are each nearby S480. While mutation of Lys522 did not affect WNG1 kinase activity, the Arg523 mutant showed a partial reduction in activity (Figure 4B).

## Discussion

**Figure 6.**
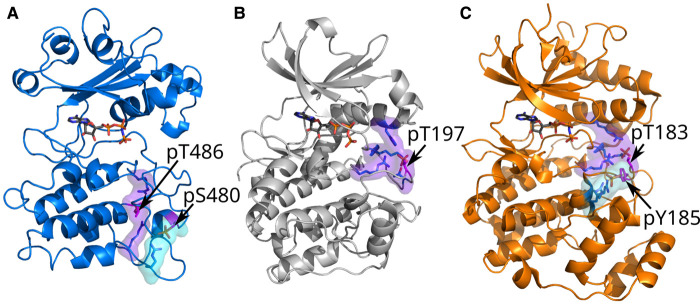
WNG1 activating phosphosite network is at a different position than canonical kinases. The activating phosphosites, shown as magenta sticks are co-ordinated by basic residues shown as blue sticks to form the phosphosite interaction network. The primary and secondary phosphosite interaction networks are represented in purple and cyan surface, respectively. (**A**) WNG1-pS480-pT486 model is represented in blue cartoon. (**B**) PKA1-pT197 (PDB: 1ATP [25]) is represented in light gray cartoon. For better visibility some portions of PKA have been omitted (**C**) ERK2-pT183-pY185 (PDB: 6OPG [57]) is represented in orange cartoon.

We have identified that the divergent kinase WNG1 appears to be activated by autophosphorylation of residues Ser480 and Thr486. Notably, WNG1 does not require phosphorylation on the canonical activation loop for activation, and Ser480 and Thr486 are located in a region of the kinase C-lobe distinct from the activating sites of canonical kinases (Figure 6). While the WNG family pseudokinase multimerizes in solution, WNG1 shows no signs of multimerization at physiologically relevant concentrations (low μM). We note, however, that WNG1 is a peripheral membrane protein [34], and we cannot rule out that such membrane association may drive interactions that are not apparent in solution.

The substrates of a canonical kinase are typically phosphorylated on flexible loops [38]. The kinase activation loop forms β-strand-like interactions with the substrate backbone to position it correctly in the active site [39,40]. For this reason, recognition of activation loop phosphorylation is often critical to docking of substrate. Our data indicate that the WNG1 HRD Arg436 does not co-ordinate the activation loop to activate the kinase as occurs in canonical kinases. Consistent with this idea, WNG1, appears to phosphorylate the majority of its substrates on or proximal to α-helices [34]. As α-helices are characterized by a completed hydrogen-bond network along the sequence [41], the backbone would not be available for interaction with the activation loop. Taken together, these data suggest that WNG1 may recognize its substrates in a manner distinct from a canonical kinase [34], though further structural work is required to define that mechanism.

Several WNG1 substrates tubulate membranes upon integration [42–44], which would be expected to be harmful to the parasite if allowed to target incorrectly, such as to the parasite Golgi or plasma membranes. How WNG1 activity is controlled *in vivo* is, therefore, an important and outstanding question. We have previously demonstrated WNG1 is inactive while trafficking through the parasite secretory system [34]. It is likely that WNG1 is maintained inactive until it comes into proximity of the PV membrane to prevent premature insertion of substrates into the parasite plasma membrane. In addition, WNG1 is primarily associated with the PV membrane after secretion, though, unlike its known substrates, the kinase is not an integral membrane protein [34].

**Figure 7.**
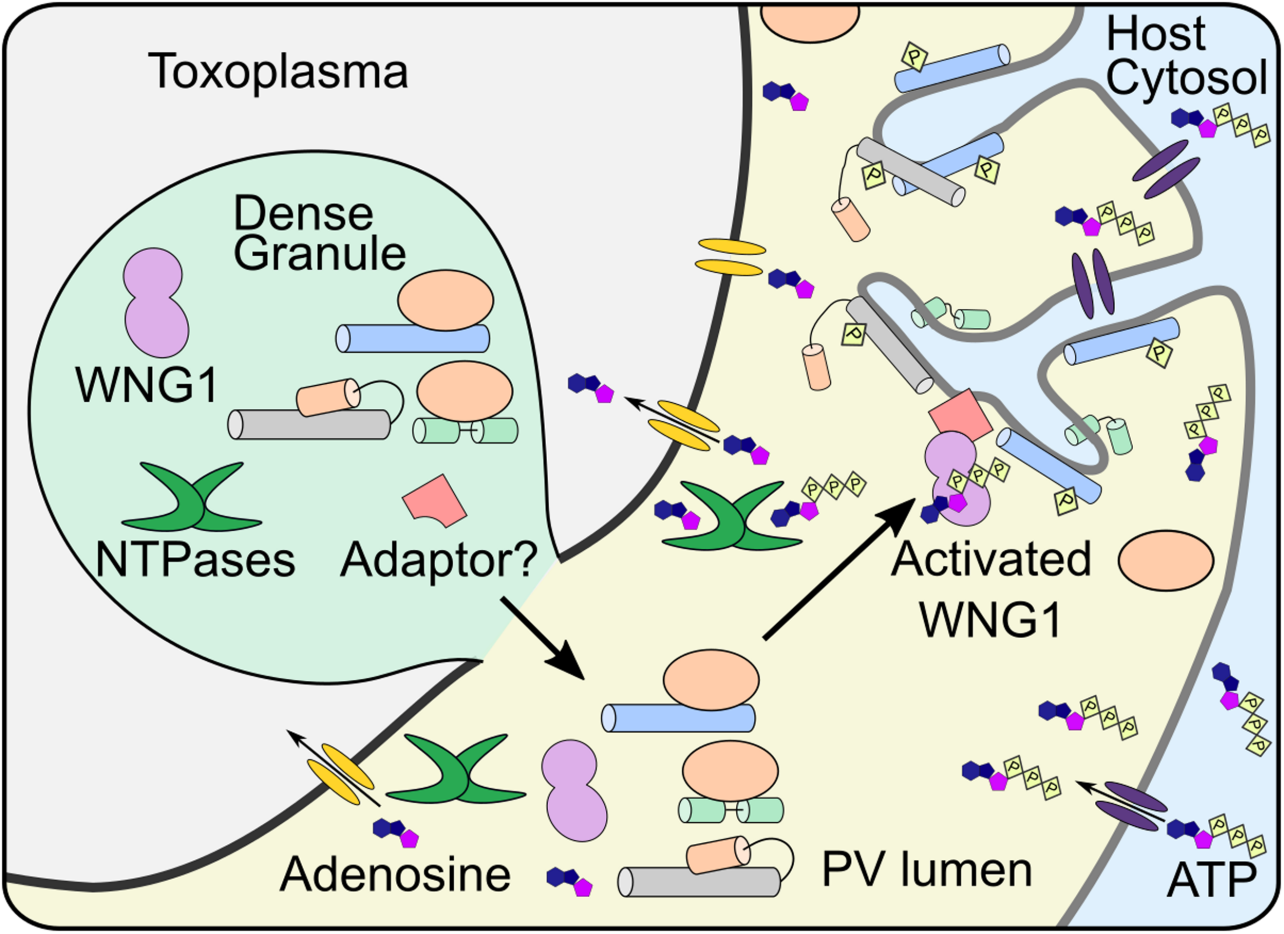
Model for activation of WNG1 *in vivo*. The dense granule contains WNG1, NTP hydrolases, other GRA proteins (blue, gray, green cylinders), their chaperone (orange) and the adaptor for WNG1 membrane integration. Upon release into the PV, the NTP hydrolases convert the ATP to adenosine for parasite uptake, reducing the ATP concentration in the lumen. The released WNG1 diffuses to the PV membrane where it gets autoactivated in the presence of high ATP levels. The activated WNG1 adheres to the PV membrane with the help of the adapter protein. Activated WNG1 phosphorylates GRAs resulting in their insertion into the PV membrane and formation of the IVN. The adenosine transport channel is represented in yellow while the ATP transporter in the PV membrane is represented in purple.

We propose a model for *in vivo* activation of WNG1 in which the major requirement in WNG1 activation is the availability of ATP (Figure 7). Recombinant WNG1 is active *in vitro* and robustly autophosphorylates, suggesting it does not require another kinase for its activation*.* Because the *K*_M,ATP_ for WNG1 (∼500 μM) is relatively high compared with most typical kinases [34], surrounding ATP levels must approach millimolar concentration for reasonable activity. The ATP concentration in the PV would be highest at the PV membrane, as ATP is thought to diffuse from the host cytosol through a pore in the PV membrane [45,46]. Regardless, ATP levels in the PV are clearly sufficient for WNG1 activity, as we have previously demonstrated [34]. However, *Toxoplasma* is auxotrophic for purines [47] and secretes highly active NTPases into the PV lumen to convert ATP to adenosine for parasite uptake at its plasma membrane [48]. Taken together, these data suggest a strong gradient of ATP concentration in the PV lumen where levels are lowest near the parasite plasma membrane and highest near pores in the PV membrane. We, therefore, propose that WNG1 autophosphorylates soon after secretion into the PV, though its activity is tuned by local ATP concentration. Because of its high *K*_M,ATP_, WNG1 would have highest activity as it approaches the PV membrane, where it becomes tightly associated with an as-yet unknown integral membrane partner. The activated WNG1 then phosphorylates GRA proteins and induces their integration into the PV membrane.

## Materials and methods

### PCR and mutagenesis

The WNG1 (265–591) and BPK1 (61–377) cDNA were previously cloned in the pET28a vector with the SUMO tag [34]. Mutagenesis was performed by Phusion QuikChange protocol [49] using Phusion polymerase (New England Biolabs). All sequences were confirmed by Sanger sequencing. The primers are listed in Supplementary Table S2.

### Protein purification

WNG1 (265–591) wild-type, mutants and BPK1 (61–377) were purified as per the protocol described in [34]. All proteins were expressed in Rosetta 2 (DE3) cells overnight at 16°C post-induction with 500 mM IPTG. The WNG1-expressing cells were resuspended in 20 mM Tris–HCl pH 7.5, 500 mM NaCl and 15 mM Imidazole (Resuspension buffer) and lysed by sonication. The lysate was centrifuged at 48k rcf and bound to Ni-NTA agarose beads (Qiagen). The beads were washed with the resuspension buffer and eluted with 20 mM Tris–HCl pH 7.5, 500 mM NaCl and 300 mM Imidazole. The elution buffer was exchanged to 20 mM HEPES pH 7.5, 300 mM NaCl, 5 mM MgCl_2_ and 10% glycerol during concentration. The concentrated protein was further purified by size exclusion chromatography (SEC) first using Sephacryl S-200 HR 16/60 and then Superdex 200 pg 16/60 columns. The final protein was concentrated and flash frozen for long-term storage in 20 mM HEPES pH 7.5, 300 mM NaCl, 5 mM MgCl_2_ and 10% glycerol buffer. For the dephosphorylated WNG1 used in Figure 2, WNG1 was coexpressed with lamda phosphatase and purified as above. The affinity chromatography for BPK (residues 61–377) protein was identical with the WNG1 protein preparation. The BPK1 protein was digested with ULP1 post Ni-NTA elution and anion exchange chromatography was performed to remove the SUMO Tag. SEC was performed as the final stage of purification using 20 mM HEPES pH 7.5, 100 mM NaCl as the buffer. The final protein was concentrated, flash frozen, and stored until further use.

### Mass spectrometry for phosphosite identification

Equal amounts of purified wild-type WNG1 and the kinase dead D437S mutant recombinant proteins were loaded on Bio-Rad Mini-PROTEAN precast gel. The gel was stained with Gel-code Blue (Thermo Fisher) and the WNG1 bands were cut out into small pieces and transferred to fresh tubes. The gel samples were digested overnight with trypsin (Pierce) following reduction and alkylation with DTT and iodoacetamide (Sigma–Aldrich). The samples then underwent solid-phase extraction cleanup with an Oasis HLB plate (Waters) and the resulting peptides were injected onto an Orbitrap Fusion Lumos mass spectrometer coupled to an Ultimate 3000 RSLC-Nano liquid chromatography system. Samples were injected onto a 75 μm i.d., 50-cm long EasySpray column (Thermo) and eluted with a gradient from 0% to 28% buffer B over 60 min at 250 nl/min. Buffer A contained 2% (v/v) ACN and 0.1% formic acid in water, and buffer B contained 80% (v/v) ACN, 10% (v/v) trifluoroethanol, and 0.1% formic acid in water. The mass spectrometer operated in positive ion mode with a source voltage of 2.2 kV and an ion transfer tube temperature of 275°C. MS scans were acquired at 120 000 resolution in the Orbitrap and up to 10 MS/MS spectra were obtained in the ion trap for each full spectrum acquired using higher-energy collisional dissociation (HCD) for ions with charges 2–7. Dynamic exclusion was set for 25 s after an ion was selected for fragmentation.

Raw MS data files were analyzed using Proteome Discoverer v2.2 (Thermo), with peptide identification performed using Sequest HT searching against the *E. coli* protein database from UniProt along with the wild-type and kinase dead sequences of WNG1. Fragment and precursor tolerances of 10 ppm and 0.6 Da were specified, and three missed cleavages were allowed. Carbamidomethylation of Cys was set as a fixed modification, with oxidation of Met, methylation of Lys and Arg, demethylation of Lys and Arg, trimethylation of Lys, acetylation of Lys and phosphorylation of Ser, Thr and Tyr set as a variable modification. The false-discovery rate (FDR) cutoff was 1% for all peptides.

### *In vitro* kinase assays

All kinases assays were conducted in technical triplicate, which were then averaged to form a single biological replicate. A minimum of three biological replicates are presented for each condition tested. The kinase assays were performed using 2 μM of SUMO-WNG1 (residues 265–591) protein, 20 mM HEPES pH 7.5, 300 mM NaCl, 5 mM MgCl_2_, 10% glycerol, 1 mM DTT, 1 mg/ml BSA and 200 μM cold ATP. γ-[P^32^]-ATP equivalent of 10 μCi and 5 mM MBP (substrate) was added at the end and incubated at 30°C for 30 min. Note that to test autophorylation of WNG1 in Figure 2A, kinase assays were conducted without BSA and with the addition of 0.1% Triton-X-100. Note that for activation experiments in Figure 2B, incubation with hot ATP and MBP was conducted for 1 min. Experiments with ‘pre-incubation’ indicate a 5 min at 30°C incubation with full reaction without hot ATP or MBP. The reaction was stopped by adding 6× SDS loading dye. The kinase activity of the proteins was visualized or quantified by running the reactions on 15% SDS–PAGE. The gel was stained with Coomassie stain and the MBP bands were cut out and the radioactivity was measured using a scintillation counter.

### Small angle X-ray scattering

Purified BPK1 protein was used for SAXS analysis at the advanced proton source (APS), Lemont, U.S.A.. The protein was diluted from a stock concentration of 305 μM in 20 mM HEPES pH 7.5, 100 mM NaCl and the scattering data were collected at the ID12 beamline. The SAXS dataset was analyzed using the ATSAS software package [50]. DAMMIN [51] was used for the construction of the *ab initio* molecular envelope. Five independent models were generated in the DAMMIN run and the theoretical I(q) was compared with the experimental I(q) for each of them. SASREF [52] from the ATSAS suite was used for making a P2 symmetry-based BPK1 tetramer. We used a monomer from the BPK1 crystal structure (PDB ID—6M7Z [34]) as the starting point for tetramer reconstruction. We compared the fits (by Chi-squared; *χ*^2^) of the SASREF-based tetramer model to the hexameric crystal structure into the DAMMIN envelope using SUPALM [53]. SAXS data were deposited in SASBDB [54] (accession: SASDNT5).

### Differential scanning fluorimetry

5 µM in 20 mM HEPES pH 7.5, 300 mM NaCl, 5 mM MgCl_2_ and 10% glycerol buffer was mixed with 10× SYPRO Orange (ThermoFisher). For a single measurement, 100 μl of the above mix was taken in a clear, non-skirted 96-well PCR plate. It was centrifuged at 800×***g*** for 5 min to remove bubbles. The fluorescence was measured from 4°C to 85°C with increments of 0.5°C in a Bio-Rad CFX96 real-time PCR machine. The melting curves for each protein was measured in triplicates. The normalized and averaged graph was plotted. The melting point was identified as the temperature at which the normalized fluorescence was 50% of the maximum.

### Size exclusion chromatography coupled with multi-angle light scattering (SEC-MALS)

2 mg/ml of WNG1 protein was injected on a Superdex 200 10/30 increase column that had been equilibrated with 20 mM Tris pH 7.0, 300 mM NaCl. 100 µL of protein was used for injection and the flow rate was 0.5 ml/min for the SEC-MALS run. The protein was detected using a Shimadzu UV detector, a Wyatt TREOS II light-scattering detector and a Wyatt Optilab T-rEX differential-refractive-index detector. Data were analyzed with Wyatt's ASTRA software version 7.1.0.29.

### Structure modeling

The WNG1 homology model was generated using BPK1 crystal structure (PDB:6M7Z [34]) as template in Modeller v9.14 [55], as described in Beraki *et al.* [34].

### Figure generation

All figures were created in Inkscape. PyMOL [56] was used for the analysis of structural models and the generation of ray-traced figures. Statistical analyses were performed in Graphpad Prism unless otherwise noted.

## Data Availability

SAXS data were deposited in SASBDB [54] (accession: SASDNT5).

## Open Access

Open access for this article was enabled by the participation of University of Texas Southwestern Medical Center in an all-inclusive *Read & Publish* agreement with Portland Press and the Biochemical Society.

## Abbreviations

GRA: dense granule proteins
MBP: myelin basic protein
PV: parasitophorous vacuole
SEC: size exclusion chromatography
SEC-MALS: size exclusion chromatography coupled with multi-angle light scattering
WNG: With-No-Gly-loop
