# Supplementary material for: Divergent kinase WNG1 is regulated by phosphorylation of an atypical activation sub-domain
Source: Biochem J. 2022 Sep 16;479(17):1877–89. doi: 10.1042/BCJ20220076 (PMC9555795; doi:10.1042/BCJ20220076)
Supplement: Supplementary Material [file BCJ-479-1877-s1.pdf]

Supplemental Materials

The WNG-family pseudokinase, BPK1, can adopt different multimeric states

One mechanism by which kinase activity can be regulated is through changes in the multimerization state. We had previously crystallized a WNG family member, the pseudokinase BPK1 (residues 61-377), and found that it forms homohexameric assembly in all crystal forms we obtained (Figure S1). Notably, the BPK1 pseudoactive sites face into the closed hexamer. Consistent with its multimerization, BPK1 has been proposed to play a structural role in *Toxoplasma* bradyzoite cyst wall formation [1]. To confirm that the multimerization state is not an artifact of crystallization, we performed small-angle x-ray scattering (SAXS) on recombinantly purified BPK1. Initial analysis of the BPK1 scatter indicated high sample monodispersity of a well-folded protein with an approximate Rg of 40 Å (Figures 1C-F, Table 1). We used the DAMMIN [2] program to obtain a molecular envelope for the most probable in-solution state of the BPK1. The resulting model showed good congruence with the experimental scattering values with a χ^2 = 1.1 (Figure 1G). While the predicted molecular envelope was much larger than a BPK1 monomer, it was not large enough to contain the BPK1 hexamer from the crystal structure. Instead, the molecular weight calculated from the volume of correlation (Vc; Table 1) was consistent with a BPK1 tetramer. Additionally, while the crystallographic hexamer fit poorly in the calculated envelope (χ^2 = 187; Figure 1H), the generated tetramer of BPK1 fit well in the molecular envelope (χ^2 = 6.2; Figure 1I). Importantly, there is no tiling arrangement by which a tetrameric state can be arranged to form closed hexamers; to form the hexameric contacts we observed in the crystal, the BPK1 tetramer must first dissociate. Therefore, BPK1 can adopt different multimerization states.

Table S1: SAXS Parameters for BPK1

Method	Parameter	
Guinier Analysis	Rg	38.49
Indirect fourier transformation	Dmax	114.55
Distance Distribution	Rg	41.9
Porod Volume (Å^3)		206804
Molar Mass determination by Vc (kDa)	Mw	148.24
Theoretical Mw of Monomer (kDa)		35.95

Figure S1

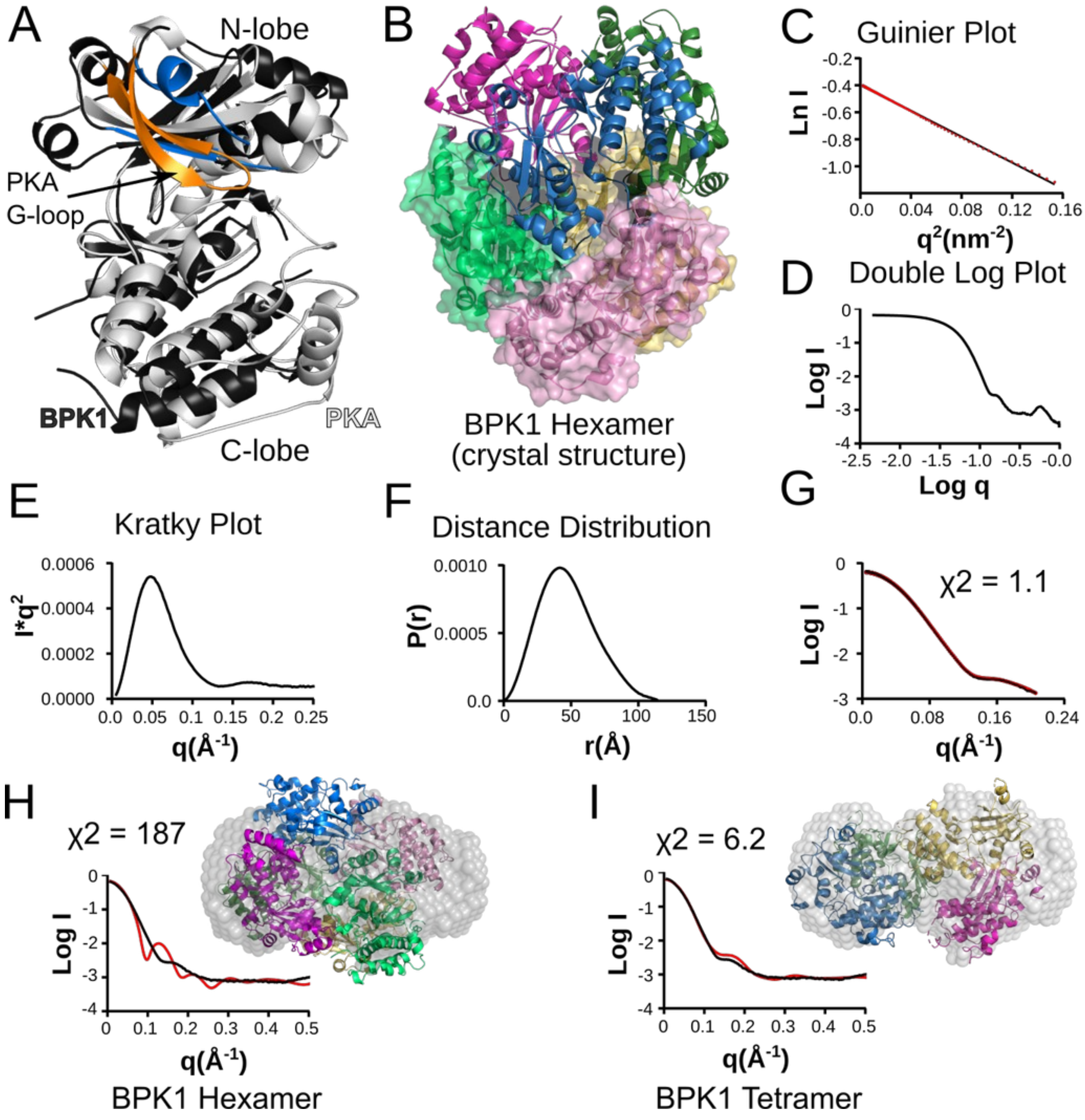

Figure S1: The WNG family pseudokinase TgBPK1 is a homo-oligomeric protein.

(A) The structures of human PKA1 (PDB – 1ATP) and TgBPK1 (PDB – 6M7Z) are represented in light gray and black cartoon respectively. The Gly-loop of PKA1 is represented in orange and the corresponding region in TgBPK1 is colored blue (adapted from [3]). (B) The asymmetric unit in the TgBPK1 crystal structure (PDB – 6M7Z; [3]. Three subunits are shown as cartoon (blue, magenta and green) while other subunits are also shown as surfaces (yellow, pink, sea green). (C) Guinier plot [Ln (q) vs q2], (D) Double log plot [Log q vs. Log I(q)], (E) Kratky Plot [I*q2 vs q] (F) pairwise distance distribution P(r) plot for TgBPK1 in-solution SAXS data analysis. (G) The calculated model (red line) from DAMMIN analysis was fitted with the experimental data (black line) with a χ2 = 1.1. The

molecular envelope generated for TgBPK1 by in-solution SAXS data analysis fitted with the TgBPK1 homohexamer (H) and homotetramer (I) with the corresponding graphs of their fit to experimental data. The tetramer showed better fit with $\chi 2 = 6.2$, while the hexamer has $\chi 2 = 187$. The individual subunits in the tetramer are shown as blue, yellow magenta and green. The hexamer color scheme is same as the (B). The black line indicate the experimental scattering data and the red line indicate the pdb based scattering generated for fitting.

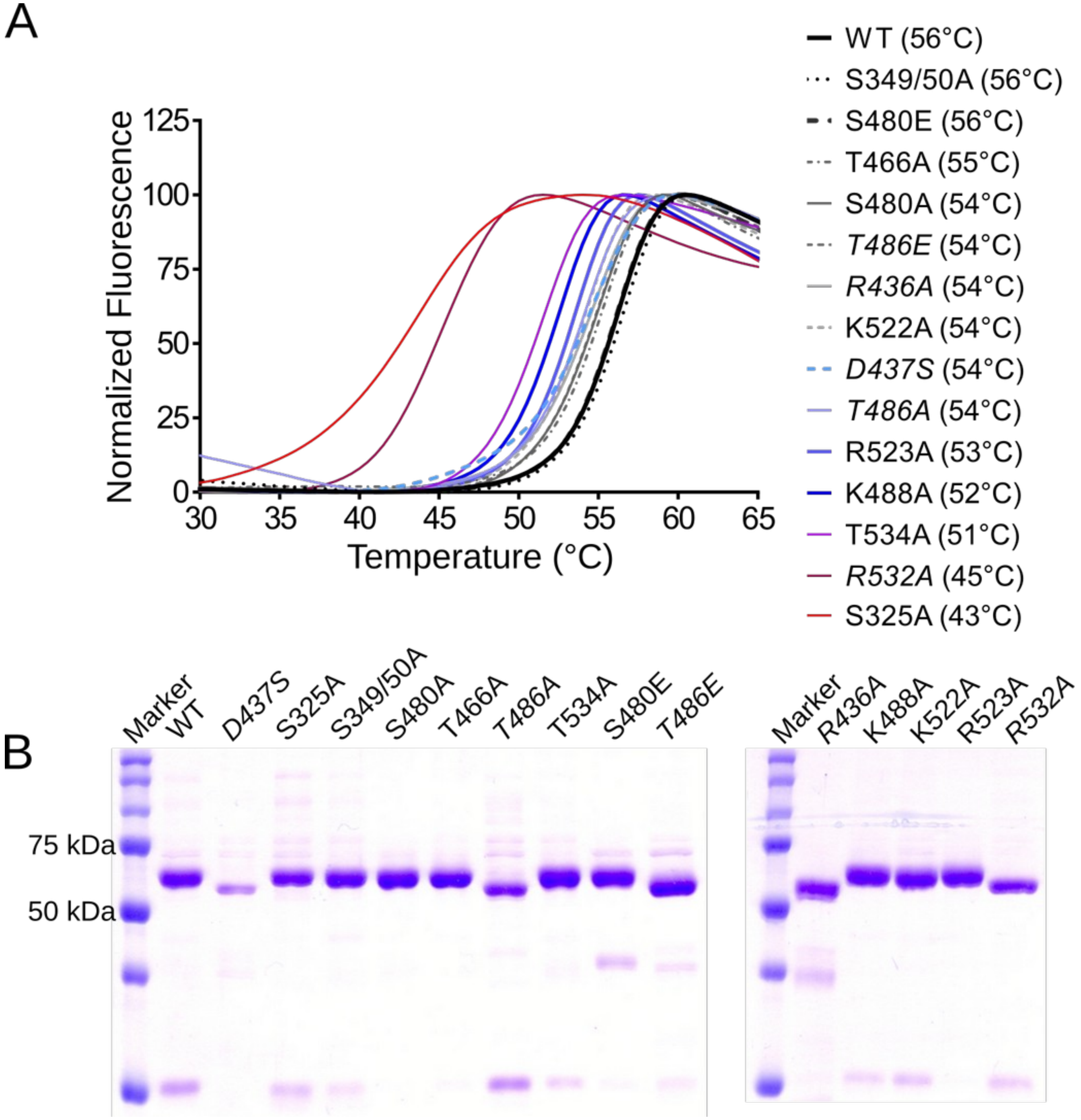

Figure S2: WNG1 activity and thermal stability are not correlated

(A) Differential scanning fluorimetric (DSF) melting profiles for WNG1 WT and mutants are shown in the graph. The graph legends include the Tm for each mutant shown in parenthesis. The inactive mutants are in italic font. (B) A 12% SDS PAGE gel showing purified WNG1 mutants used in the DSF study. All the inactive mutants run lower in the gel due to absence of autophosphorylation.

Table S2: Primers used for WNG1 mutagenesis

WNG1 (265-591) Mutant	Primer	Sequence (5'->3')
S325A	Forward	GCCGAGGCGCTGGCGGAC
S325A	Reverse	CACGTGCGGCACGCGC
S349/50A	Forward	gctgCAACGGACGCTGAGGCGCG
S349/50A	Reverse	CCGATGCAGGAAGCCGTGTGTAT
S480A	Forward	GCGCGGGCCACTGACCACAC
S480E	Forward	GAGCGGGCCACTGACCACAC
S480A/E	Reverse	CAGTTCCGGGGCGAAGTATCC
T466A	Forward	GCCCCCGTCGTCGGCACCCG
T466A	Reverse	GCGCTGCTGGAGCACGCC
T486A	Forward	gCAGAGAAATCCGATGTCTTCGC
T486E	Forward	GAAGAGAAATCCGATGTCTTCGC
T486A/E	Reverse	GTGGTCAGTGGCCCGCGAC
T534A	Forward	gCGCTGAAACAGGTCATGGAAG
T534A	Reverse	CGGCCGCTCCTCTGGATC
R436A	Forward	CATGCGGACATCAAGGCTCACAAC
R436A	Reverse	CAGGAAGCCGTGTGTATGGAGAAT
K488A	Forward	GCGTCCGATGTCTTCGCTCTTGGC
K488A	Reverse	CTCTGTGTGGTCAGTGGCCCG
K522A	Forward	GCGCGGATGACCGCGAAAGATC
K522A	Reverse	AGTGAGGGCCCAGAGCTCG
R523A	Forward	GCGATGACCGCGAAAGATCCAGAG
R523A	Reverse	CTTAGTGAGGGCCCAGAGCTC
R532A	Forward	GCGCCGACGCTGAAACAGGTCATG
R532A	Reverse	CTCCTCTGGATCTTTCGCGGTCATC

Supplemental References

1 Buchholz, K. R., Bowyer, P. W. and Boothroyd, J. C. (2013) Bradyzoite pseudokinase 1 is crucial for efficient oral infectivity of the Toxoplasma gondii tissue cyst. Eukaryot. Cell **12**, 399–410.

2 Svergun, D. I. (1999) Restoring Low Resolution Structure of Biological Macromolecules from Solution Scattering Using Simulated Annealing. Biophys. J. **76**, 2879–2886.

3 Beraki, T., Hu, X., Broncel, M., Young, J. C., O’Shaughnessy, W. J., Borek, D., Treeck, M. and Reese, M. L. (2019) Divergent kinase regulates membrane ultrastructure of the Toxoplasma parasitophorous vacuole. Proc. Natl. Acad. Sci., National Academy of Sciences **116**, 6361–6370.
